# Retraction Note: Effect of intranasal instillation of *Escherichia coli* on apoptosis of spleen cells in diet-induced-obese mice

**DOI:** 10.1038/s41598-024-52877-9

**Published:** 2024-01-31

**Authors:** Zhihua Ren, Xuchu Gu, Jing Fang, Dongjie Cai, Zhicai Zuo, Shuang Liang, Hengmin Cui, Junliang Deng, Xiaoping Ma, Yi Geng, Ming Zhang, Yue Xie, Gang Ye, Liping Gou, Yanchun Hu

**Affiliations:** Key Laboratory of Animal Disease and Human Health of Sichuan Province, College of Veterinary Medicine, Sichuan Agricultural University, Sichuan Province, Chengdu, 611130 China

Retraction of: *Scientific Reports* 10.1038/s41598-020-62044-5, published online 20 March 2020

The Editors have retracted this Article at the Corresponding Authors' request. After publication, concerns were raised regarding the images presented in Fig. 3, specifically:

• Fig. 3 Lean-*E. coli* 0 h, DIO-*E. coli* 24 and 72 h images appear to overlap;

• Fig. 3 Lean-*E. coli* 12 and 24 h images appear to overlap.

The Authors have been unable to provide the underlying raw data upon request. The Editors therefore no longer have confidence in the presented data.

All Authors agree to this retraction.

